# Self-help management of patients undergoing chemotherapy: analysis of the online forum of the women’s self-help association against cancer

**DOI:** 10.1007/s12282-023-01481-2

**Published:** 2023-07-07

**Authors:** C. Colditz, C. Keinki, J. Huebner

**Affiliations:** Klinik Für Innere Medizin II, Universitätsklinikum Jena, Am Klinikum 1, 07747 Jena, Germany

**Keywords:** Breast cancer, Gynecologic cancer, Chemotherapy, Digital forum, Patient information

## Abstract

**Purpose:**

The aim of this study was to examine threads on chemotherapy in the largest German self-help forum regarding content and emotions.

**Methods:**

All threads on the subject of chemotherapy that were published by February 6th, 2022 were included in the category “drug therapy”. A total of 50 threads were analyzed. A quantitative analysis was carried out with regard to content, emotions, number of replies, number of hits, duration of the conversation, duration of access in days, number density of replies, and hits per day.

**Results:**

16 threads are about side effects and in 18 threads, the emotion is fear. Threads in which the emotion fear was expressed have the highest number of replies at 3367. Shared therapy successes are posted with pleasure and achieved a higher mean value for the duration of conversation with 1374.25 days.

**Conclusion:**

An online self-help forum is a very important source of psychosocial support for patients undergoing chemotherapy.

## Introduction

Medical topics are becoming increasingly popular when it comes to online research. It is mostly women who look online for advice on medical issues [1]. It is particularly helpful for patients with cancer to gather experiences and tips from other patients online [2]. They usually find support in online groups or by e-mail [1]. All of this helps to better adjust emotionally to the disease. Sharing experiences reduces the sense of isolation that comes with the diagnosis [2]. Online groups are now organized in large organizations with both local and online representation [1].

Only in online self-help forums are there such a wealth of different experiences that you can bring into discussions with other participants. This creates a feeling of community. In contrast to this are messenger services and short messages. Only individual thoughts are shared here and no question is asked and discussion is not encouraged [3].

Patients can log into online forums at any time without having to inform anyone. They do not have to tell something about themselves online if they do not want to. You can share your story or stay completely anonymous. Everyone can make this decision for themselves. In this way, they can express their feelings openly and honestly without causing any emotion that may negatively affect the relationship to relatives or friends or having the fear of being compromised. These support groups are always available, even when traditional sources such as doctors are not. Questions can be asked here that remained unanswered or are not sufficiently clarified at the time the diagnosis was made [4]. The knowledge gained from such online offers is helpful to ensure that patients receive good advice during therapy [5].

With the diagnosis of the disease, different needs for emotions and information arise in the patients. In digital forums people are not alone with these needs, but are understood and these needs are met. Relationships are formed with other users based on the diagnosis of the same disease [6].

These self-help groups are mostly used for three reasons: participants want to manage living with the disease, are looking for support, or are looking for answers to questions about their therapy [7].

The women's self-help cancer group is the best-known self-help community for women with cancer in Germany. It was founded in 1976 by an initiative of 16 women to change the shortcomings in breast cancer care at this time. The organization grew exponentially in the following years and is now represented throughout Germany. Around 35,000 people are looked after. The German Cancer Aid takes over the patronage for the women's self-help cancer as well as the financial support [8].

The organization's self-help forum went online for the first time in 2013, and the number of users has also increased exponentially since it was launched [8]. Today the forum has around 7000 members. Online forums play an important role as a source of information, but can also lead to negative emotions or the spread of false information [9]. To prevent this and to ensure proper and respectful interaction between members, the forum is run by various moderators.

The aim of this study was to better understand how online self-help groups work in relation to drug therapy for cancer with special attention given to chemotherapy. We aimed at analyzing the contributions of participants in such a network with respect to content and emotions exchanged in the group.

## Materials and methods

### Material

The digital forum for women’s self-help was founded in 2013. Access to the threads is free for everyone. At the time of data collection, 6963 members were registered and there were 467,929 posts on 7798 topics. The forum is run by volunteers. As moderators, they pay attention to, among other things, the passing on of false information and the way participants interact with each other. On the main page of the forum there is a division into “General topics” (introduction round, encouragement, stories, announcements and dates) and “Our topics” (types of cancer, social issues, who is like me?, how do I help myself?, treatment methods).

For the analysis, the category “drug therapy” was selected from the treatment methods in the digital self-help forum. All threads related to chemotherapy, chemotherapy drugs, side effects, therapy, experiences with the therapy medication, therapy success, treating doctors, knowledge about chemotherapy or inpatient vs. outpatient chemotherapy were included. All threads not related to chemotherapy in any way were excluded. All articles on chemotherapy that were published by February 6th, 2022 were included in this category.

### Extraction

Data acquisition and evaluation was initially carried out using Microsoft Excel.

All posts related to chemotherapy have been transferred to Excel with the link and title of the conversation.

A quantitative analysis of all first posts in a thread was carried out with regard to content, title of the conversation, emotion, number of replies, number of hits, start date of the conversation, the date of the last post by February 6th, 2022, named chemotherapeutic agents or therapies, the duration of the conversation in days, duration of access until February 6th, 2022 in days, number of answers per day, and number of accesses per day. The next step was a detailed analysis of answers in a thread that either provided new content or revealed emotional reactions to the topic. Answers simply agreeing or disagreeing to aforementioned content or emotions were skipped. A maximum of 10 replies per thread were included in the evaluation. The selected answers were transferred to Excel with full text. The answers were evaluated according to content, emotion and author.

For a detailed analysis, we chose the methodology by Mayring [10]. First one author (CC) read all threads and coded the main topic, the expressed emotion and the most meaningful answers. The most meaningful answers are the ones with the most important content and emotional aspects for the first author of the thread. The coding is based on a content analysis of the first thread and the selected answers. In the next step a second author (JH) also coded the threads using the wording of the codes of the first authors provided in a list and adding additional items if appropriate. In the last step, both authors agreed on the coding list and categories that result from the coding and a comparison of the coding was done. In case of differences, these were solved by discussion.

Then the top 20 posts were evaluated quantitatively according to number of replies, number density of replies per day, number of hits, number density of hits per day and length of conversation in days. In addition, the classification of the respective contribution was recorded.

This was followed by the quantitative evaluation of the content from the respective first post according to absolute frequency, total number of replies, total number of hits, average length of conversation in days, average number density of answers per day and average number density of hits per day. The evaluation of the emotions expressed from the first posts followed the same pattern.

### Analysis and statistics

Following this, the data of the quantitative analysis were transferred to the 28.0.1.1 (14) version of SPSS. For this, the content and emotions were replaced with variables. Then it was checked how often an emotion was expressed for the respective topic. The asymptotic significance (*p* = 0.033) of the correlations was checked with Pearson's chi-square tests.

### Ethics vote

According to the statutes of the university hospital Jena and because no studies were carried out on humans, no ethics vote was required for this study.

## Results

A total of 50 threads with a maximum of 10 replies were systematically analyzed. Of this, 39 threads were written by patients undergoing chemotherapy. A total of 6 threads were started by relatives. 3 relatives wrote contributions for their mothers, 1 for their sister, 1 for their wife and 1 for their girlfriend. In the case of 5 threads, the subject was not clear.

### Topics of the threads

**Fig. 1:**
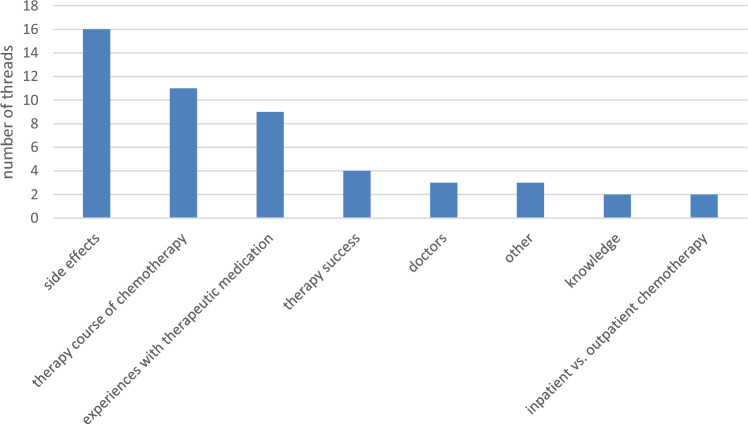
Topics of the threads (*n* = 50)

A total of 16 threads were related to side effects of chemotherapy, 11 addressed the course of chemotherapy, 9 were related to experiences with the therapy medication, 4 therapy successes, 3 discussed problems with doctors, 2 contained knowledge, 2 the topic of inpatient vs. outpatient chemotherapy. The last 3 threads included other topics (see Fig. 1).

### Emotions in the threads

**Fig. 2:**
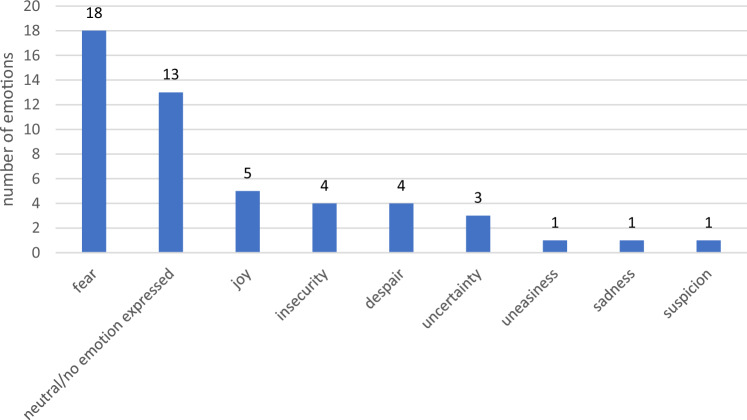
Emotions of the threads (*n* = 50)

Emotions were clearly shown in most threads. Clear emotions were expressed in 37 threads. 8 emotions vary in the threads. Fear was expressed in 18 threads. In 13 others, the mood was neutral or no emotion was expressed. Joy came up in 5 threads, and uncertainty and despair in 4 threads each. Uncertainty was expressed in 3 threads and 1 thread each contained concern, distress and suspicion (see Fig. 2). Negative emotions clearly dominate the threads.

The median conversation duration was 766 days (range 1–2826 days), the median number of replies was 107.82 (range 0–2100), and the median number of hits was 26,021.9 (range 439–445,000). The mean number of replies per day was 0.76 with a range of 0–8.5 and the mean number of hits per day was 16.51 with a range of 0.91–244.91.

The thread entitled “Xeloda—Experiences (Capecitabine)” has the highest number of replies with 2100, the highest number of hits with 445,000 and the highest number density of hits per day with 244.1. This thread was written about side effects with the emotion fear.

The thread with the title “When does the chemo work?” has the most answers per day with a value of 8.5. This thread also expresses the emotion of fear. The content is the course of chemotherapy.

The thread titled “Erbulin (Halaven) Therapy Experience?” has the longest active runtime with a score of 2826 days. The emotion of fear is also expressed here.

The threads on side effects are far ahead of the other threads with a total of 3257 replies. Likewise, these threads have the largest sum of number of accesses with 797,224 and the highest average number of accesses per day with 27.6. Threads on therapy success achieved a higher mean value for the duration of conversation with 1374.25 days and those on the course of chemotherapy with 1.58, a higher mean value for the number density of responses per day than the threads on side effects.

In contrast, threads on the topic of inpatient vs. outpatient chemotherapy have the lowest sum of responses at 45, the lowest mean number density of responses per day at 0.06, and the lowest mean number density of accesses per day at 4.06. Knowledge threads have the lowest total hits at 8200 and the lowest mean conversation length in days at 33.

Threads in which the emotion fear was expressed have the highest number of replies at 3367, the clearly highest number of hits at 851,426 and the highest mean number density of hits per day at 28.88. These threads get the most attention. Threads related to happiness have significantly fewer replies (934), hits (177,200) and hits per day (15.83).

### Comparison of the threads

Threads on the subject of therapy success are 100% linked to the emotion of joy. In contrast, threads in which knowledge is imparted are 100% neutral or no emotion is expressed. When it comes to the decision of inpatient vs. outpatient chemotherapy, the emotion fear is expressed 100%.

Dealing with doctors is clearly characterized by negative feelings with 66.7% in fear and 33.3% in insecurity. The users do not understand why they are getting a certain therapy or treatment. They do not know who decides about the therapy or treatment. These circumstances cause great insecurity and distrust in the treating doctors.

When it comes to obtaining experience with therapy medication, no emotion is expressed in 44.4% of cases. The other threads are characterized by negative feelings such as fear (22.2%), uncertainty (11.1%), insecurity (11.1%) and uneasiness (11.1%).

When it comes to side effects, the feeling of fear is expressed at 37.5% of the threads. In 18.8%, no or a neutral emotion is expressed. Likewise, 18.8% express despair. Joy, sadness, suspicion and insecurity are expressed as emotions at 6.3% each.

During the course of chemotherapy, the absolute majority (54.4%) expressed fear. At 27.3% no emotion is recognizable. A percentage of 9.1% are despaired in relation to the course of therapy (see Table 1).

**Table 1:** Relationship between content and emotion

Classification content * classification emotions Crosstabulation												
			Classification emotions									Total
			Neutral/no emotion expressed	Fear	Sadness	Joy	Despair	Suspicion	Insecurity	Uncertainty	Uneasiness	
Classification content	Therapy success	Count	0	0	0	4	0	0	0	0	0	4
		% within classification content	0.0%	0.0%	0.0%	100.0%	0.0%	0.0%	0.0%	0.0%	0.0%	100.0%
	Experiences with therapeutic medication	Count	4	2	0	0	0	0	1	1	1	9
		% within classification content	44.4%	22.2%	0.0%	0.0%	0.0%	0.0%	11.1%	11.1%	11.1%	100.0%
	Side effects	Count	3	6	1	1	3	1	1	0	0	16
		% within classification content	18.8%	37.5%	6.3%	6.3%	18.8%	6.3%	6.3%	0.0%	0.0%	100.0%
	Therapy course of chemotherapy	Count	3	6	0	0	1	0	0	1	0	11
		% within classification content	27.3%	54.5%	0.0%	0.0%	9.1%	0.0%	0.0%	9.1%	0.0%	100.0%
	Knowledge	Count	2	0	0	0	0	0	0	0	0	2
		% within classification content	100.0%	0.0%	0.0%	0.0%	0.0%	0.0%	0.0%	0.0%	0.0%	100.0%
	Inpatient vs. outpatient chemotherapy	Count	0	2	0	0	0	0	0	0	0	2
		% within classification content	0.0%	100.0%	0.0%	0.0%	0.0%	0.0%	0.0%	0.0%	0.0%	100.0%
	Doctors	Count	0	2	0	0	0	0	1	0	0	3
		% within classification content	0.0%	66.7%	0.0%	0.0%	0.0%	0.0%	33.3%	0.0%	0.0%	100.0%
	Other	Count	1	0	0	0	0	0	1	1	0	3
		% within classification content	33.3%	0.0%	0.0%	0.0%	0.0%	0.0%	33.3%	33.3%	0.0%	100.0%
Total		Count	13	18	1	5	4	1	4	3	1	50
		% within classification content	26.0%	36.0%	2.0%	10.0%	8.0%	2.0%	8.0%	6.0%	2.0%	100.0%

The differences in emotions between the different subjects are significant (*p* = 0.033). Thus, the content and the expressed emotion are related.

## Discussion

The analysis shows that there is a wide range of topics related to chemotherapy in the forum. The respective topics are associated with strong, clear emotions. Most threads concern side effects of chemotherapy. The most strongly represented emotion is the fear regarding chemotherapy.

Chemotherapy side effects threads received the most replies (3257), most hits (797,224), and most hits per day (27.6). In terms of the duration of the conversation, the side effects only took fourth place with 881.75 days. The majority of these threads (37.5%) are associated with the emotion of fear. All but one of the other threads are related to negative emotions. Only in 1 thread is the side effect of joy expressed.

These threads receive a lot of attention and interest in the forum, answers are given quickly and the participation of other users is high. Negative emotions strongly attract the attention of the forum. With 3367 answers, fear is the emotion that is reacted to the most. Previous research has also shown that seeking emotional support in online self-help forums plays an important role [11].

Therapy successes also receive a great deal of attention. They receive a total of 822 replies and 152,200 hits. They have the longest duration of conversations (1374.25 days). All threads related to this topic are linked with joy. Success in chemotherapy receives a lot of attention and there is a lot of encouragement and joy from other participants. It is of great importance for everyone to highlight and celebrate the successes and also not to neglect the positive events in therapy.

Posts on the course of chemotherapy receive the most responses per day (1.58) and are the second most common post with an absolute frequency of 11. In these posts, fear dominates at 54.5%. The participants are afraid of what to expect and how everything will turn out. There is also uncertainty and despair. Except for three posts on this topic, all are associated with negative emotions. The other three are written neutrally and no emotion is expressed. The unequivocal dominance of negative emotions clearly shows what a major break chemotherapy triggers in the emotional world of the patient. It has previously been shown that psychosocial well-being is one of the main intentions of users of an online self-help forum [12]. Most users express understanding for the situation of others and share tips from their own experiences [13]. The shared information is usually a combination of their own experiences and the knowledge of the treating physician [14].

Threads about experiences with therapy medication are the third most common threads on chemotherapy with an absolute frequency of 9. A total of 44.4% of these posts show no emotion or are written neutrally. The participants objectively ask about the experiences of other participants. Most frequently, questions are asked directly about experiences or side effects of certain medications that were newly prescribed due to a change in therapy. Participants want to gather the knowledge and experiences of others in order to be prepared for what might happen to them. They obtain the knowledge for their respective medication in a targeted manner [15]. If an emotion is expressed, then it is exclusively negative (fear, insecurity, insecurity, worry). Previous research has shown that sharing information helps patients cope better with their diagnosis and the side effects of therapy [16].

Contributions in which only knowledge is imparted are always written neutrally and are not linked to any emotion. It’s all about educating and informing, for example about treatment options. These threads get few replies and hits, and conversations are the shortest. At 1.2 responses per day, they receive the second most common number of responses per day. This placement is due to the very short duration of the conversations. The number of replies and hits and the neutral nature of the posts shows that these posts play a small role when it comes to chemotherapy in the forum.

Threads dealing with inpatient versus outpatient chemotherapy decisions receive the fewest replies (45), have the fewest replies per day (0.06), and have the fewest hits per day (4.06). All threads on this are linked to the emotion of fear. Patients find it difficult to assess the pros and cons of the different chemotherapy options themselves.

Three contributions deal with the attending physicians of the participants. Only negative emotions are expressed here: 66.7% fear and 33.3% uncertainty. Due to a lack of communication with the patient or insufficient education, most patients become afraid and seek advice online. Previous studies have shown that, from the patients' point of view, their questions were not sufficiently discussed with the doctors and not enough supportive therapy options were given [17]. The Internet plays a major role in processing and understanding physician information. Patients want to use the Internet to better understand their illness and look for possible treatment options [2]. Unsatisfactory doctor-patient communication is a major motivator to search the internet for answers [15].

### Limitations

As the evaluation was limited to one online self-help forum of the women’s association against cancer, generalizability to other genders or patients not organized in self-help may not be given and other analyses would be helpful to fill in this gap. Moreover, only the posts on chemotherapy up to February 6th, 2022 were included. Also the selection of the answers and the limit of 10 answers per thread may lead to a bias in data. Coding by two persons also may lead to a bias, even if these two persons have quite a different background (CC being an advanced medical student and JH having a professorship for integrative oncology). While coding of content most probably is less prone to bias, coding of emotions may be less stable by different researchers. Using an established model of emotions as that of Plutchik was tested by us in a former study. Yet, neither that model nor any other we tried offered the range of emotions we found so that we decided to develop the wording appropriate for the data we found in the threads. Last but not least, our analysis is mostly quantitative. A qualitative analysis would provide more insight into the content and emotions.

## Conclusion

Many cancer patients are faced with chemotherapy during their treatment. A feeling of fear usually sets in with a view to the upcoming chemotherapy. This is often associated with unanswered questions about side effects of the therapy. Only a few threads directly address the insecurity and fear of the treating physicians.

The self-help forum offers a good opportunity to better manage one's own illness and life with it independently, it enables support from others and for others in similar situations and it offers the opportunity to clarify open questions about one's own therapy or to answer other questions [7].

A forum where questions can be asked or experiences shared is a great support for the lengthy and difficult chemotherapy. Joy and sorrow are shared here with others. Treatment successes are celebrated in the forum and bad news is expressed with sympathy and helpfulness. For a large group of patients, it is very important to establish contact and a relationship with others suffering from the same disease [6].

An online self-help forum is an important source of psychosocial support for patients and it should be pointed out more often, especially by treating physicians. Online self-help forums provide an opportunity to meet these needs in addition to the help of treating physicians, family and friends. The results of this work may include information for doctors and nurses to improve their communication with patients. Moreover, they may look for well-moderated fora and recommend them to patients actively. It can also have a positive effect on educating patients about additional information and exchange opportunities.

## Data Availability

The datasets generated during and/or analysed during the current study are available from the corresponding author on reasonable request.
